# The emerging contribution of social wasps to grape rot disease ecology

**DOI:** 10.7717/peerj.3223

**Published:** 2017-04-26

**Authors:** Anne A. Madden, Sean D. Boyden, Jonathan-Andrew N. Soriano, Tyler B. Corey, Jonathan W. Leff, Noah Fierer, Philip T. Starks

**Affiliations:** 1 Biology Department, Tufts University, Medford, MA, United States; 2 Department of Ecology and Evolutionary Biology, University of Colorado at Boulder, Boulder, CO, United States; 3 Cooperative Institute for Research in Environmental Sciences, University of Colorado at Boulder, Boulder, CO, United States; 4 Current affiliation: Keck Center For Behavioral Biology, North Carolina State University, Raleigh, NC, United States; 5 Current affiliation: Department of Applied Ecology, North Carolina State University, Raleigh, NC, United States; 6 Current affiliation: Cue Biopharma, Cambridge, MA, United States; 7 Current affiliation: School of Biological Sciences, University of Nebraska, Lincoln, NE, United States

**Keywords:** Sour rot, *Aspergillus niger*, Summer rot, Bunch rot, Hornet, Yeast, Acetic acid bacteria, *Polistes dominula*, Polymicrobial disease, *Vitis vinifera*

## Abstract

Grape sour (bunch) rot is a polymicrobial disease of vineyards that causes millions of dollars in lost revenue per year due to decreased quality of grapes and resultant wine. The disease is associated with damaged berries infected with a community of acetic acid bacteria, yeasts, and filamentous fungi that results in rotting berries with high amounts of undesirable volatile acidity. Many insect species cause the initial grape berry damage that can lead to this disease, but most studies have focused on the role of fruit flies in facilitating symptoms and vectoring the microorganisms of this disease complex. Like fruit flies, social wasps are abundant in vineyards where they feed on ripe berries and cause significant damage, while also dispersing yeasts involved in wine fermentation. Despite this, their possible role in disease facilitation and dispersal of grape rots has not been explored. We tested the hypothesis that the paper wasp *Polistes dominulus* could facilitate grape sour rot in the absence of other insect vectors. Using marker gene sequencing we characterized the bacterial and fungal community of wild-caught adults. We used a sterilized foraging arena to determine if these wasps transfer viable microorganisms when foraging. We then tested if wasps harboring their native microbial community, or those inoculated with sour rot, had an effect on grape sour rot incidence and severity using a laboratory foraging arena. We found that all wasps harbor some portion of the sour rot microbial community and that they have the ability to transfer viable microorganisms when foraging. Foraging by inoculated and uninoculated wasps led to an increase in berry rot disease symptom severity and incidence. Our results indicate that paper wasps can facilitate sour rot diseases in the absence of other vectors and that the mechanism of this facilitation may include both increasing host susceptibility and transmitting these microbial communities to the grapes. Social wasps are understudied but relevant players in the sour rot ecology of vineyards.

## Introduction

Sour rot (bunch rot) is one of the most important diseases that affects wine grape quality worldwide (Barata et al., 2011; Steel, Blackman & Schmidtke, 2013). This polymicrobial disease involves acetic acid bacteria, Ascomycota yeasts, and filamentous fungi that attack ripe, thin-skinned cultivars in the late summer (Barata, Malfeito-Ferreira & Loureiro, 2012a; Nally et al., 2013). Infection leads to berry decomposition, the overgrowth of contaminating saprophytic yeast species, acetic acid bacteria resulting in the production of acetic acid, and in some cases, if certain strains of *Aspergillus* species are present, accumulation of carcinogenic mycotoxins such as Ochratoxin A (Bisiach, Minervini & Zerbetto, 1986; Zoecklein, Williams & Duncan, 2000; Varga & Kozakiewicz, 2006; Barata et al., 2008). Collectively this results in a negative impact on wine quality. There is no effective treatment for sour rot. Current management strategies rely on reducing environmental conditions that facilitate sour rot, the use of chemical fungicides to control prior fungal infections that facilitate sour rot development (UC IPM Pest Management Guidelines: Grape, 2014), and the reduction of known vectors (Bisiach, Minervini & Zerbetto, 1986).

Sour rot microorganisms are part of the normal microbiota of grape berry skins (Barata, Malfeito-Ferreira & Loureiro, 2012a), but the development of disease symptoms typically requires further interactions with insects (Barata et al., 2012). While many insects damage ripe grape berries, fruit flies (*Drosophila melanogaster*) have received particular attention for their role in sour rot. Fruit flies vector sour rot microorganisms and are implicated in making the host more susceptible to infection (Barata et al., 2012). As described by others (Barata et al., 2012), fruit flies are intuitive subjects for such studies based on their ecology: they are abundant in vineyards, they are attracted to the aroma of sour rot infected (fermenting) berries (Becher et al., 2012), they carry live yeasts and bacteria on and in their body (Coluccio et al., 2008), and they are capable of transferring these microorganisms among fruits. All of these attributes suggest that fruit flies may have a role in sour rot ecology; however these attributes are not unique to fruit flies.

Wasps (family: Vespidae) inhabit orchards and vineyards in the late summer, where they become locally abundant (Watkins et al., 1979). Though primarily carnivorous throughout the early part of their colony cycle, in the late summer they forage on sugar-rich food like ripe grape berries (Matsuura & Yamane, 1990). In doing so, they are often considered vineyard pests, as they harass grape pickers and produce significant berry damage that subsequently attracts disease vectors (Watkins et al., 1979; Cranshaw, Larsen & Zimmerman, 2011). Like fruit flies, they harbor viable yeast (Stefanini et al., 2012) and are attracted to microbial volatiles such as those produced by rot-associated organisms (Landolt et al., 1999; Landolt et al., 2014; Davis, Boundy-Mills & Landolt, 2012). Unlike fruit flies, wasps have additional traits that could facilitate microbial dispersal, such as the ability to forage over greater distances of tens to hundreds of meters per foraging trip (Dew & Michener, 1978), and the ability of adults to directly injure grape berries with their strong mandibles (Mani, Shivaraju & Kulkarni, 2014). Despite these characteristics, wasps have received little attention for the role they may have in the microbial ecology of plant diseases such as berry rot.

Here we tested the hypothesis that the invasive paper wasp, *Polistes dominulus*, can facilitate grape sour rot in the absence of other vectors. We used foraging experiments in lab enclosures to understand the effect of wasps on the development of grape rot symptoms. We further explored the relationship of these wasps with sour rot microbial dispersal by testing the ability of *P. dominulus* to vector microorganisms while foraging. Finally, we used culture-independent methods to characterize the bacterial and fungal communities of *P. dominulus* to determine if they carry the sour rot disease complex in their environment.

## Methods

### Disease facilitation experiments

To determine if *P. dominulus* foraging facilitated incidence or severity of sour rot in grape berries we performed two sets of foraging experiments: one where wasps were inoculated with a sour rot community and one where they were not inoculated and harbored only their native community.

### Disease facilitation experiment 1. Inoculated *P. dominulus* foragers with injured and uninjured grapes

Our first experiment assessed whether *P. dominulus* inoculated with a sour rot community could facilitate disease in damaged and undamaged berries. We used methods developed by others to assess sour rot vectoring by fruit flies (Barata et al., 2012). Briefly, 156 white, seedless table grape berries (*Vitis vinifera* L, cultivar Thompson Seedless) purchased from Whole Foods Market were separated and washed. Berries were ripe at the start of the experiment (median pH = 4.0). Per Barata et al. (2012), we washed each berry in a 10% bleach solution followed by a 70% ethanol solution. We modified the methods of Barata et al. (2012) in that the only source of rot microorganisms was inoculated insects, not the inoculated insects *and* inoculated grape skins. To assess if prior damage was required for wasp disease facilitation we aseptically damaged half the grapes to replicate natural berry splitting. This is a phenomenon that occurs in vineyards due to water pressure imbalances in the berries after heavy rain events, or in berries that have densely packed clusters (Dean et al., 2016). Six grape berries were randomly selected from either the uninjured, or injured group and placed in an autoclaved 1 L glass enclosure. We employed a fully factorial design for grape injury and wasp presence with six enclosures per treatment. All enclosures included six grapes (either injured or uninjured), and six or zero wasps.

We collected 72 *P. dominulus* adults from multiple populations in eastern Massachusetts. *P. dominulus* contain highly variable microbial communities (A Madden, 2017, unpublished data), therefore we supplemented these wasps with a mixture of a simplified sour rot community: the yeast *Schizosaccharomyces pombe* (Carolina Biologicals, Burlington, NC, USA), and the acetic acid bacterium *Acetobacter aceti* ATCC 15973™ (American Type Culture Collection, Manassas, Virginia). These species have previously been isolated from sour rot infected grapes (Barata, Malfeito-Ferreira & Loureiro, 2012a). To inoculate the wasps, *A. aceti* was grown in glucose yeast extract medium and *Sc. pombe* was grown in glucose yeast extract peptone medium at 25 °C to dense cultures of OD_600_ 1.7. We then placed each adult in a small receptacle for 18 h that contained equal volumes of yeast and bacteria (approximately 500 μl of bacterial and 500 μl of yeast culture) (Fig. S1). The wasps were inoculated externally and internally when they performed ecologically relevant feeding and grooming behaviors (Sumana & Starks, 2004).

Following wasp additions, all enclosures were maintained for 13 days on a natural day/night light cycle at ∼23 °C, following the approximate timeline of the assay developed by Barata et al. (2012). During this time, the wasps foraged on the grapes as they do in vineyards (Fig. S2). Some wasps died during the assay period, as expected based on previous demographic work on a similar wasp species (Reeve et al., 1998). Any wasps that died during the treatment incubation time were not removed from, or replaced in, the enclosures. None of the wasps that died during the duration of the assay showed any indication of microbial infection, or filamentous fungal growth that would influence grape berry rot.

On the 13th day of the assay, we removed all grapes from the enclosures and photographed them. These photographs were assessed for disease incidence and berry decomposition severity by two independent observers blinded to treatment. Berry decomposition severity was assessed using a quality scale (0–4) (Fig. S3). As many of the grapes had an abundance of black spores on them, black mold incidence was quantified as the percentage of berries per enclosure with visible filamentous fungal growth and spores. Scores were averaged across observers. To determine if berry damage was necessary for disease development we statistically compared black mold incidence and berry decomposition scores of uninjured grapes with and without wasps using the Mann–Whitney *U* test. Because there was no effect of wasps on uninjured berries, all further measurements and statistical comparisons were made solely with injured berries (+∕ − wasps).

To determine if wasps had an effect on black mold incidence in injured grapes, we compared the average percentage of grapes showing symptoms per enclosure using a Mann–Whitney *U* test. To determine if wasps had an effect on disease severity we compared berry decomposition scores of injured grapes with and without wasps across enclosures using a Mann–Whitney *U* test.

Greater berry decomposition, and higher acetic acid and gluconic acid concentrations from acetic acid bacteria are the distinguishing characteristics of sour rot (Bisiach, Minervini & Zerbetto, 1986; Barata et al., 2012). We therefore calculated acetic acid concentration per berry as a second metric of disease severity. We measured acetic acid concentration of the four most diseased berries in the injured grape berry treatments with and without wasps. We determined the concentration of grape acetic acid using the Megazyme Acetic Acid kit, per the manufacturer’s protocol (Megazyme International, Bray, Co. Wicklow, Ireland). We compared acetic acid concentration of these grapes across enclosures for those grapes with and without wasps using a Mann–Whitney *U* test.

### Filamentous fungus pathogen identification

In many of the grapes in the presence of foragers, and a few without, we noticed considerable black spores associated with a filamentous fungus. To identify the fungus responsible for this symptom, we isolated representative strains of the fungus and sequenced the ITS1-5.8S-ITS2 gene fragments. Strains were isolated from grapes. DNA of the strains was extracted using the PowerSoil^®^ microbial DNA extraction kit (MO BIO, Carlsbad, CA) following the manufacturer’s protocol, but substituting axenic biomass for soil. The ITS1-5.8S-ITS2 rRNA gene fragments of the isolates were amplified using the universal primers Pn3 (CCGTTGGTGAACCAGCGGAGGGATC) and Pn34 (TGCCGCTTCACTCGCCGTT), following the amplification protocol of Viaud, Pasquier & Brygoo (2000). The PCR cocktail (25 µl) contained 5.0 µl DNA template, 12.5 µl GoTaq MasterMix™ (Promega, Madison, WI), and 1.0 µM of each primer. The PCRs were performed in a 2700 GeneAmp PCR System (Applied Biosystems, San Francisco, CA) for 30 cycles of 95 °C for 60 s, 50 °C for 90 s, and 72 °C for 60 s. Successful amplicons were visualized by electrophoresis and purified using the UltraClean^®^ DNA purification kit (MO BIO, Carlsbad, CA) per the manufacturer’s protocol. Purified amplicons were sent for Sanger sequencing to MacrogenUSA (Cambridge, MA) using the amplification primers. A consensus contiguous sequence for each isolate was constructed using DNA Baser Sequence Assembler v3.5.4 software (Heracle BioSoft S.L.R. Pitest, Romania). Putative fungal identities were then determined using the BLAST algorithm (Altschul et al., 1990) and the GenBank database.

### Disease facilitation experiment 2. Uninoculated *P. dominulus* foragers with injured grapes

In a second foraging experiment, we determined if wasps could facilitate grape berry disease when they harbored their native microbial communities. We performed the assay as described above, but with uninoculated wasps. These wasps were collected from eastern Massachusetts near the locations of previously collected wasps. We further modified the foraging experiment described above in a number of ways to assure that we were only measuring grapes in the experimental group that the wasps had foraged on: (1) We used two wasps per one injured grape per enclosure with associated controls. (2) We measured berry decomposition score and acetic acid as described above on day zero and then on the final 12th and 13th days of the experiment. (3) Unlike the previous experiment, replicates were only included if both wasps survived at least four days of the assay. This resulted in 5 replicates for treatments on day zero, and 10 replicates without wasps and 6 replicates with wasps at the conclusion of the assay. Total disease scores and acetic acid concentrations were statistically compared with controls at the start and conclusion of the assay with Mann–Whitney *U* tests.

#### Dispersal capability experiment 1: characterization of sour microbial complex in adult *P. dominulus*

As part of a separate study, we characterized the total bacterial and fungal community of adult *P. dominulus* (A Madden, 2017, unpublished data). To determine if uninoculated *P. dominulus* harbor the sour rot microbial complex we looked at 49 adults from this study that were from five populations in eastern Massachusetts. Briefly, we homogenized the adult bodies and extracted DNA using a modified manufacturer’s protocol (Fierer et al., 2008). We amplified the bacterial 16S and fungal ITS1 DNA using 515f and 805r (covering the v4 region of the 16S) and the ITS1-F and ITS2 amplification primers (respectively). The primers contained illumina adapters and the reverse primers included unique 12 base pair barcodes allowing for multiplex sequencing. Amplification was carried out using the cycling parameters of 94 °C for 5 min, with 40 cycles of 94 °C for 45 s, 50 °C for 30 s, and 72 °C for 90 s, followed by a final extension at 72 °C for 10 min (for each targeted amplicon) (Barberán et al., 2015). All samples were run in triplicate with a no template control. Successful amplicons were cleaned using the Mobio Ultra-Clean™ PCR clean-up kit. DNA concentration was assessed fluorescently, using the Invitrogen Quant-IT PicoGreen^®^ dsDNA assay kit (Carlsbad, USA). DNA concentrations were normalized across samples and pooled (individually for bacteria and fungi). Sequencing was performed in multiplex on two runs of a MiSeq using 2 × 150 bp chemistry (separate runs for 16S and ITS amplicons). Sequences (paired end reads for bacteria and reverse reads only for fungi) were processed using the UPARSE pipeline (Edgar, 2013). We used the RDP classifier for taxonomy assignments, with methods and reference databases described elsewhere (Barberán et al., 2015). Non-target sequences (e.g., mitochondria and chloroplasts) were removed prior to downstream processing (see Madden et al., In Prep for more details of the generation and processing of amplicon sequences). To estimate the relative abundances of the sour rot microorganisms and to control for differences in sequencing depth, sequences were subsampled at 1990 sequences per wasp for bacteria and 50 for fungi. As this reduced the number of sequences and samples analyzed, we additionally used all sequences from samples when determining if a sour rot microorganism was detected in a wasp. Because of this unequal sampling, detection of any sour rot microorganism should be interpreted as presence-only, not presence-absence data. The bacterial and fungal taxa associated with sour rot were identified from literature reports (Barata, Malfeito-Ferreira & Loureiro, 2012a; Barata et al., 2012; Nally et al., 2013). These included acetic acid bacteria (family: Acetobacteraceae), and Ascomycota yeasts (class: Saccharomycetes). A group of saprophytic filamentous fungi (*Aspergillus* spp., *Penicillium* spp*.*, *Botrytis* spp*.*, *Cladosporium* spp*.*, and *Alternaria* spp*.*) were additionally analyzed as these taxa are frequently associated with the development of sour rot symptoms (Rooney-Latham et al., 2008; Barata, Malfeito-Ferreira & Loureiro, 2012a; Barata, Malfeito-Ferreira & Loureiro, 2012b).

#### Dispersal capability experiment 2: microorganisms shed by *P. dominulus* when foraging

To determine if wasps could disperse viable microorganisms when foraging we made a sterile arena where we could detect the abundance of microorganisms dispersed to plants by visiting wasps. This arena included sterilized pseudoflowers that contained sugar water to motivate foraging (Fig. S4). After allowing foragers to visit the pseudoflowers during a 24-hour period, the pseudoflower petals were imprinted on microbial growth media to detect viable microorganisms transferred. Following incubation we counted the number of microbial colony forming units that grew on paired forager and forager-excluded pseudoflowers (Fig. S5). We replicated this assay 11 times with 11 unique nests and associated foragers. We compared the number of dispersed microorganisms in each trial using a paired *t*-test (See Fig. S4 for more details on methods and assay validation).

## Results and Discussion

Sour rot causes millions of dollars in losses to vineyards annually (Steel, Blackman & Schmidtke, 2013), yet we know little about the ecology of this disease, including what insects are relevant to the dispersal and establishment of the sour rot microbial community. Using data from multiple foraging experiments and pairing this with data on the microbial community of *P. dominulus*, we found that paper wasps can directly influence sour rot and black mold disease (*Aspergillus* rot) in the absence of additional vectors and that they may be vectoring this microbial community to grapes when foraging.

### Rot facilitation by *P. dominulus*

#### Inoculated wasps facilitated rot in injured berries

**Figure 1:**
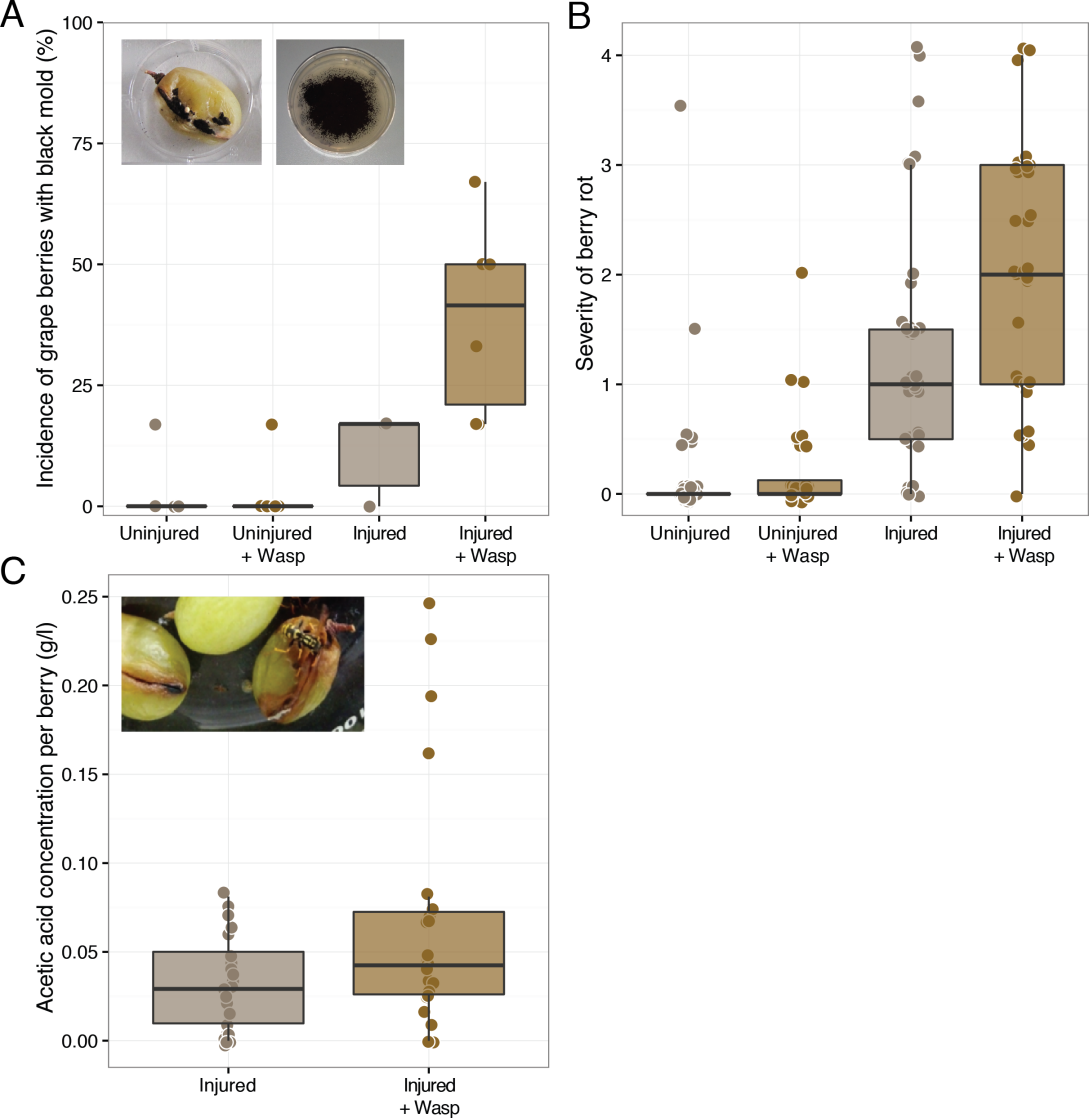
Effect of sour rot microorganism inoculated wasps on grape berry disease. Treatments include either sound (“Uninjured”) or sliced (“Injured”) grape berries in the presence of wasps (“+Wasp”:) or in the absence of wasps. (A) Wasp foraging increased the incidence of filamentous fungi, *Aspergillus* rot (black mold), presence on injured grapes (*p* = 0.02), but not uninjured berries (*p* > 0.05). (B) Wasp foraging increased the severity of disease in injured (*p* = 0.02), but not uninjured berries (*p* > 0.05). (C) Grape berries with wasps did not have higher concentrations of acetic acid on average (*p* > 0.05).

Wasp foraging led to a greater incidence and severity of rot symptoms in injured grape berries when the wasps were previously inoculated with a simplified sour rot microbial community. Injured grapes in enclosures with these wasps had greater disease scores than those injured berries exposed to air (Mann–Whitney *U*, W = 434.5, *p*-value = 0.02, *n* = 36) (Fig. 1), with more extensive symptoms of rotting, browning, and visual fungal growth (Fig. S6). Wasps are attracted to many of the volatile chemicals produced by fermenting microorganisms (Landolt et al., 1999; Davis, Boundy-Mills & Landolt, 2012). In accordance with this we often observed the wasps feeding on the most diseased of the six berries in each enclosure. This suggests that the observed effect of disease facilitation by wasps is a conservative estimate, as we are likely averaging these disease scores over those berries that had and had not come into contact with wasps.

During the later days of the experiment, the enclosures with rotten berries had the distinct smell of vinegar—the hallmark indicator of sour rot infections in vineyards (Steel, Blackman & Schmidtke, 2013). This smell is caused by the production of acetic acid and gluconic acid by the acetic acid bacteria of the sour rot complex. Despite these observations, injured grapes in the presence of wasps did not have significantly higher acetic acid concentrations than those in the absence of wasps (Mann–Whitney *U*, W = 203.5, *p*-value > 0.05, *n* = 24) (Fig. 1). While wasps did not significantly elevate average grape acetic acid concentrations, the highest acetic acid concentrations were associated with grapes in the presence of wasps (Fig. 1). Thus, grapes showing sour rot disease symptoms were associated with the presence of foraging wasps.

It is likely that the discrepancy in disease rot severity and acetic concentration arises from a secondary infection of some of the sour rot infected grapes, rather than the lack of a sour rot infection. Filamentous fungi and spores were observed on many of the injured grapes with wasps and in a few of the injured grapes without wasps (Fig. 1). Wasp foraging increased the incidence of this disease symptom on injured grapes (Mann–Whitney *U*, W = 4, *p*-value = 0.02, *n* = 6). This was somewhat surprising, as none of the microorganisms fed to these wasps result in such growth. Sequence analysis of isolates from these berries revealed that the fungus was a black mold, *Aspergillus niger* (Table S1). *As. niger* is a common pathogen of grape berries and a frequent associate of sour rot disease (*UC IPM Pest Management Guidelines: Grape*, 2014), where it metabolizes many of the organic acids in grapes, including acetic acid (Halliwell & Walker, 1952). Beyond assisting with berry decomposition, *As. niger* can produce the mycotoxin Ochratoxin A (Tjamos et al., 2004), which can contaminate resulting wine. The presence of this fungus could explain the low levels of acetic acid we observed at the conclusion of the assay, as this fungus could be metabolizing the acetic acid produced by the sour rot infection (see below). *As. niger* is often found within surface sterilized grape berries such as the ones we used in this experiment (Passamani et al., 2012), thus it was likely that the original berries selected for this experiment were already harboring *As. niger* within them. The increase in the presence of a black mold (*Aspergillus* rot) in the presence of wasps suggests that, like fruit flies (Barata, Malfeito-Ferreira & Loureiro, 2012b), wasps are facilitating berry disease by making injured host tissue more susceptible to infection.

#### Sour rot inoculated wasps did not facilitate rot in uninjured berries

Fruit flies facilitate sour rot, but are only capable of causing the disease in previously injured grapes (Barata et al., 2012). In our foraging experiment, *P. dominulus* similarly did not have an effect on grape disease severity or incidence unless the grapes were previously injured (Mann–Whitney *U*, W = 4, *p*-value = 0.02, *n* = 6, Mann–Whitney *U*, W = 18, *p*-value >0.05, *n* = 6) (Fig. 1, Fig. S6). This was somewhat surprising, as these wasps are known to cause initial damage to various fruits including grapes (*V. vinifera*) and sweet cherries (*Prunus avium*) (Cranshaw, Larsen & Zimmerman, 2011). A possible explanation for the difference between our results and field observations could be explained by the wasps’ inability to break through the skin of certain thick-skinned grape cultivars such as those used in this experiment (Galvan, Koch & Hutchison, 2008).

#### Uninoculated wasps facilitated rot in injured berries

**Figure 2:**
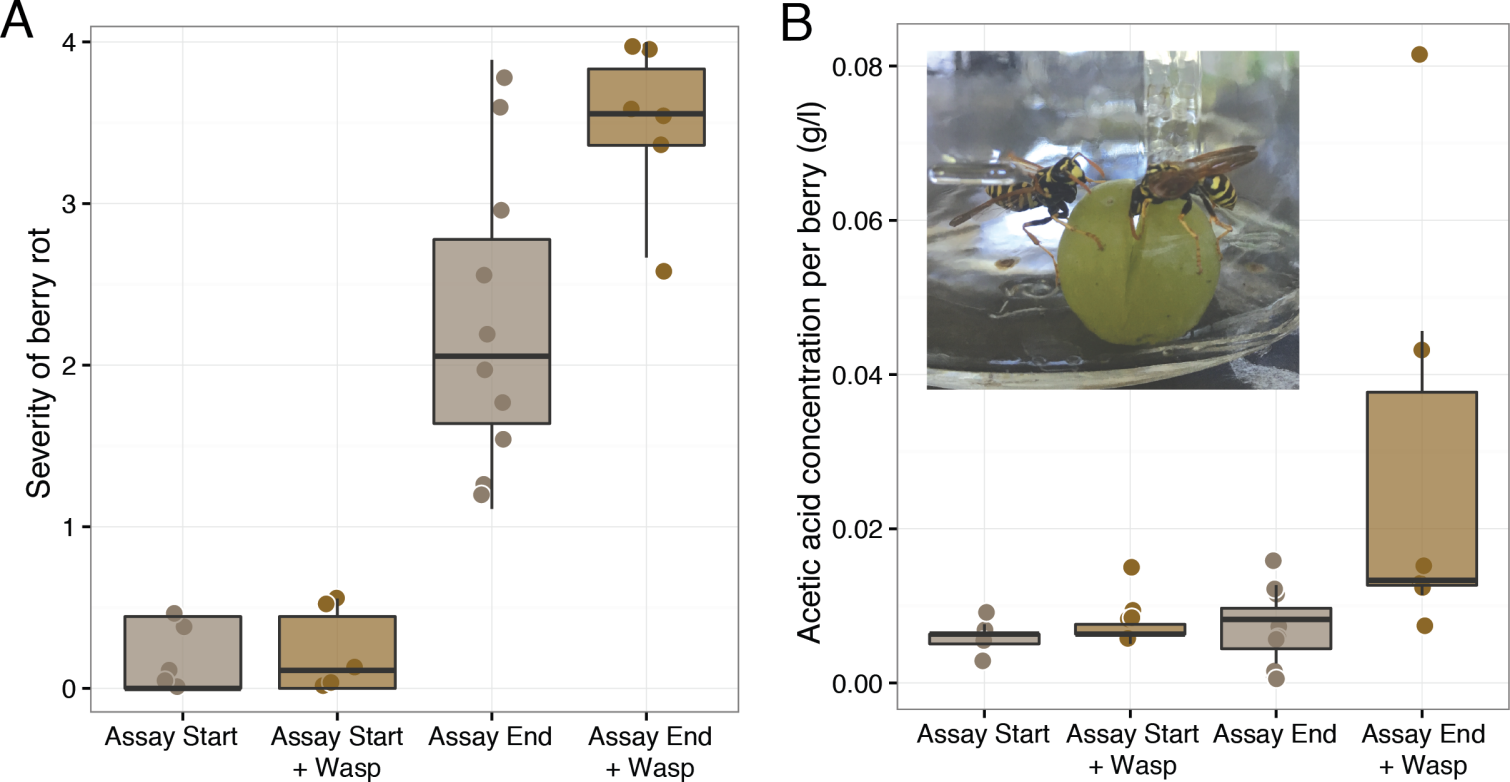
Effect of uninoculated wasps on grape berry disease. Treatments included injured grape berries plus wasps (“+Wasp”) or without wasps. Disease measurements were taken on day zero of the experiment (“Assay Start”) and day 12 and 13 (“Assay End”.) (A) Wasp foraging increased the severity of disease in injured berries (*p* = 0.03). (B) Wasp foraging increased the average concentration of acetic acid in berries (*p* < 0.01).

The results of our second foraging assay revealed that *P. dominulus* harboring only its native microbial community facilitated grape sour rot. As in the first experiment, wasp foraging led to more severely diseased grapes (Mann–Whitney *U*, W = 9, *p*-value = 0.03, controls: *n* = 10, wasp treatment: *n* = 6) (Fig. 2). In this experiment, grape berries in the presence of these wasps had higher acetic acid concentrations, indicative of sour rot (Mann–Whitney *U*, W = 2.5, *p*-value <0.01, controls: *n* = 10, wasp treatment: *n* = 6) (Fig. 2). To the best of our knowledge, this is the first study to show that wasps carrying their native microbial community can facilitate sour rot in the absence of other vectors.

Unlike in the first foraging experiment, we detected no symptoms of *As. niger* infection in these grapes. The lack of these symptoms and the higher acetic acid concentration relative to controls are consistent with our hypothesis that the *As. niger* infection may have obscured some of the sour rot symptoms in the first experiment.

### Rot dispersal by *P. dominulus*

#### Uninoculated wasps are associated with sour rot microorganisms

The sour rot microbial complex includes yeasts, filamentous fungi, and acetic acid bacteria, but the acetic acid bacteria are responsible for the worst of the disease symptoms (Barata et al., 2012). All inoculated wasps we surveyed contained acetic acid bacteria, including *Gluconobacter* spp., the genus associated with acetic acid production in sour rot infections (Barata et al., 2012). Nearly half (52%) of these wasps also carried sour rot associated yeasts, the second component of the sour rot community. Saprotrophic filamentous fungi such as *Aspergillus* spp, *Penicillium* spp., *Alternaria* spp., *Cladosporium* spp., and *Botrytis* spp. may be associates of the sour rot complex, or they may be secondary infections associated with sour rot (Barata, Malfeito-Ferreira & Loureiro, 2012a). Of the wasps we sampled, 88% carried at least one of these fungi. Of particular note, many of these wasps contained sequences from *Aspergillus* spp. and *Botrytis* spp. which are also associated with a black mold (*Aspergillus* rot) and grey mold (respectively) (Barata, Malfeito-Ferreira & Loureiro, 2012a).

**Figure 3:**
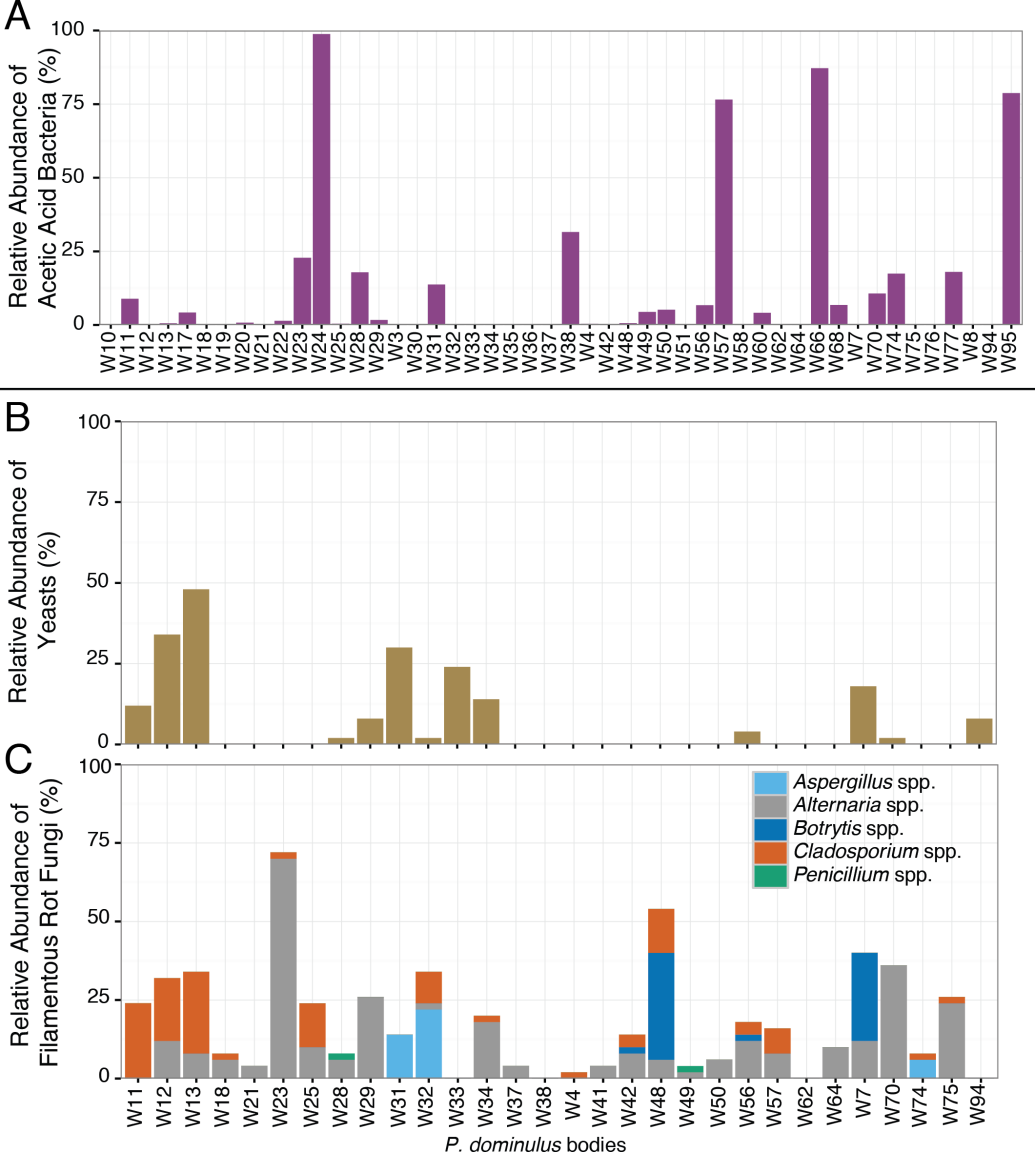
Relative abundance of sour rot associated microorganisms in *P. dominulus*. Each bar represents a unique adult. (A) Acetic acid bacteria (Acetobacteriaceae), (B) yeast (Saccharomycetes) and (C) filamentous fungi associated with rot, not limited to sour rot (*Aspergillus* spp., *Penicillium* spp., *Cladosporium* spp., *Botrytis* spp., *Alternaria* spp.). (A) Is percentage of total bacterial sequences per wasp and (B) and (C) are percentages of total fungal sequences per wasp.

While half the wasps carried the full complement of sour rot microorganisms, they carried highly variable amounts of these species (Fig. 3). In some wasps, the bacterial community was dominated by acetic acid bacteria—making up 99% of the bacterial sequences in one wasp—while in others it was barely detectable (Fig. 3). This variability is similar to that found in fruit flies, where some fruit flies harbor yeasts or acetic acid bacteria and others do not (Barata et al., 2012). This variability suggests that rather than being obligate associates of wasps, these microorganisms may be acquired from the local environment.

#### Uninoculated wasps transfer microorganisms to plants when foraging

Paper wasps have been implicated in the dispersal of microorganisms to plants, most notably yeast dispersal onto grape berries in vineyards (Mortimer & Polsinelli, 1999; Stefanini et al., 2012). However, few studies have provided evidence that wasps can transfer viable microorganisms when foraging. To test the hypothesis that *P. dominulus* disperses microorganisms while foraging, we designed a sterile arena with sterile ‘pseudoflowers’ where petals were separated from the nectar food source. This setup allowed us to test whether wasps shed viable microorganisms when performing natural foraging behaviors.

Foraging wasps dispersed significantly more microorganisms to flowers than were deposited by air, with wasps dispersing 40% more microbial propagules within 24 h than were dispersed by air alone (paired *t*-test, *t* = 7.12, *df* = 10, *p* < 0.0001) (Fig. S7). These estimates are conservative, however, as we used conditions that would select for only a portion of the transferred microbial community. In our assays, wasps were interacting with the pseudoflowers as they would interact with flowers when foraging for nectar. When wasps feed on grape berries, they are further interacting with the host tissue by walking on it, masticating pieces of it, and defecating on it (Video S1). In this assay we only looked at the effect of walking on the host tissue as part of feeding. Even with these conservativemethods, we found that wasps disperse microorganisms to host grapes and that these microorganisms are viable regardless of wasp cuticular antimicrobial secretions (Turillazzi et al., 2006).

#### Insights into the role of *P. dominulus* in grape rot ecology

Sour rot, like many rot infections, is a disease that manifests when there are the appropriate environmental conditions, a susceptible host, and the presence of a pathogen. Fruit flies are known to facilitate sour rot by increasing host susceptibility in damaged grapes, and by potentially increasing the pathogen presence by vectoring these microorganisms from their body to grapes (Barata et al., 2012). We found that foraging *P. dominulus* facilitated sour rot and an associated *Aspergillus* rot disease in grapes. The increase in disease symptoms, particularly of *Aspergillus* rot in surface sterilized grape berries suggests that wasps are facilitating the disease by making the host more susceptible to infection (Barata et al., 2012). The mechanism for this facilitation is currently unknown, but may relate to mechanical disruption of the berry tissue that elicits a hypersensitive response (Lattanzio, Lattanzio & Cardinali, 2006). However, given that we have shown these wasps can disperse microorganisms when foraging, that they are capable of causing sour rot in surface sterilized berries, and that all of the wasps we surveyed contained part or all of the microbial community associated with sour rot, it is likely that these wasps are *also* vectoring these rot microorganisms. Some of these microorganisms may directly cause the symptoms we saw (high acetic acid concentrations), or create environments that foster secondary infections (e.g. *Aspergillus* rot). Our results are remarkably consistent with those for fruit flies, suggesting *P. dominulus* may have a similar ecological role to *D. melanogaster* in sour rot ecology.

Our foraging assays were conducted in the lab to assure that no other insect vector was influencing disease symptoms. They further featured surface sterilized berries to better understand the insect origin of pathogenic microorganisms. These represent different conditions than experienced in the field, where berries host a plethora of microbial species, including those associated with various rots (Barata, Malfeito-Ferreira & Loureiro, 2012a; Gilbert, Van der Lelie & Zarraonaindia, 2014). While this likely makes the results of our foraging experiments highly conservative, future work will be needed to understand the relationship *P. dominulus* has with dispersing and facilitating sour rot in the context of vineyards. Such studies should include other wasps found in vineyards, as the relationship between the sour rot microbial community and grapes likely extends beyond *P. dominulus* to other insects with similar ecological roles. Vineyards contain many vespids which feed on grape berries (Mussen & Rust, 2012). Some of these wasps reach much higher abundances in vineyards than *P. dominulus* and have stronger mandibles capable of damaging even thick-skinned fruit (Alford, 2014).

Carnivorous wasps are rarely considered as disease vectors; however, the absence of data should not indicate the absence of such a role. The paper wasp *Polybia rejecta* facilitates fungal disease when it forages on Red-Eyed Tree Frog, *Agalychnis callidryas*, egg masses (Hughey et al., 2012). The German wasp, *Vespula germanica*, has been implicated in causing bacterial infections of udders when it forages on cows for protein (Yeruham, Schwimmer & Brami, 2002). It is therefore likely that wasps represent an important and relatively unexplored member in the multitrophic interactions of certain microbial infections.

## Conclusion

Sour rot causes millions of dollars in losses per year for vineyards, yet there are few effective management strategies or treatments. To better manage this disease we need to understand its ecology, including the multitrophic interactions that result in disease symptoms. Previous studies have revealed the importance of fruit flies as vectors and disease facilitators, but they have failed to consider the importance of other insects. We investigated the role of a social wasp on grape berry sour rot. Using foraging studies, and multiple metrics of disease severity and incidence we have shown that paper wasps can facilitate disease in previously damaged grapes. Furthermore, these wasps harbor the polymicrobial community of sour rot and are capable of dispersing live microorganisms when foraging. This suggests that wasps have a further role in vectoring this disease in vineyards. Ultimately, this work highlights that additional insects in vineyards may be playing an important role in the ecology of sour rot disease.

##  Supplemental Information

10.7717/peerj.3223/supp-1 Supplemental Information 1 Supplemental Figures, Tables, and Video Legend Figure S1. Inoculation of *P. dominulus* with the sour rot community.
Figure S2. Sour rot inoculated *P. dominulus* foraging on grape berries in the rot dispersal assay.
Figure S3. Qualitative guide used to assess overall grape berry disease score.
Figure S4. Schematic of sterile foraging enclosure to assess microbial dispersal and detailed methods.
Figure S5. Dispersal assay methodology and representative dispersed microbial growth.
Figure S6. Representative trials of grape berries maintained in the presence or absence of sour rot inoculated *P. dominulus* for 13 days.
Figure S7. Percent increase in dispersed viable microbes on pseudoflowers visited by foraging wasps relative to forager-excluded pseudoflowers.
Video S1. Supplemental video of uninoculated wasps foraging on grape berries
Table S1. Identification of fungal strains isolated form grape berries with black mold.

## Additional Information and Declarations

### DNA Deposition

The following information was supplied regarding the deposition of DNA sequences: Sequences and associated metadata generated as part of this study are available on the FigShare digital repository: https://figshare.com/s/9de094698ff26a34ec88 and in the GenBank database, accession nos. KF737862– KF737865. Supplemental video file: Madden, Anne (2017): Madden_etal_Video.mov. figshare.
https://doi.org/10.6084/m9.figshare.4551145.v1.

### Data Availability

The following information was supplied regarding data availability: FigShare digital repository: https://figshare.com/s/9de094698ff26a34ec88
doi: 10.6084/m9.figshare.4312604. Supplemental Video file available at: Figshare: https://figshare.com/s/b2d32e53a8e363c88616
doi: 10.6084/m9.figshare.4551145.
