# Supplementary material for: The emerging contribution of social wasps to grape rot disease ecology
Source: PeerJ. 2017 Apr 26;5:e3223. doi: 10.7717/peerj.3223 (PMC5408719; doi:10.7717/peerj.3223)
Supplement: Supplemental Information 1 — Figure S1. Inoculation of P. dominulus with the sour rot community. Figure S2. Sour rot inoculated P. dominulus foraging on grape berries in the rot dispersal assay. Figure S3. Qualitative guide used to assess overall grape berry disease score. Figure S4. Schematic of sterile foraging enclosure to assess microbial dispersal and detailed methods. Figure S5. Dispersal assay methodology and representative dispersed microbial growth. Figure S6. Representative trials of grape berries maintained in the presence or absence of sour rot inoculated P. dominulus for 13 days. Figure S7. Percent increase in dispersed viable microbes on pseudoflowers visited by foraging wasps relative to forager-excluded pseudoflowers. Video S1. Supplemental video of uninoculated wasps foraging on grape berries Table S1. Identification of fungal strains isolated form grape berries with black mold. [file peerj-05-3223-s001.docx]

**Madden et al. “**The emerging contribution of social wasps to grape rot disease ecology”

**Supplemental** **Information (Figures, Video, Table)**

**Supplemental Figures**


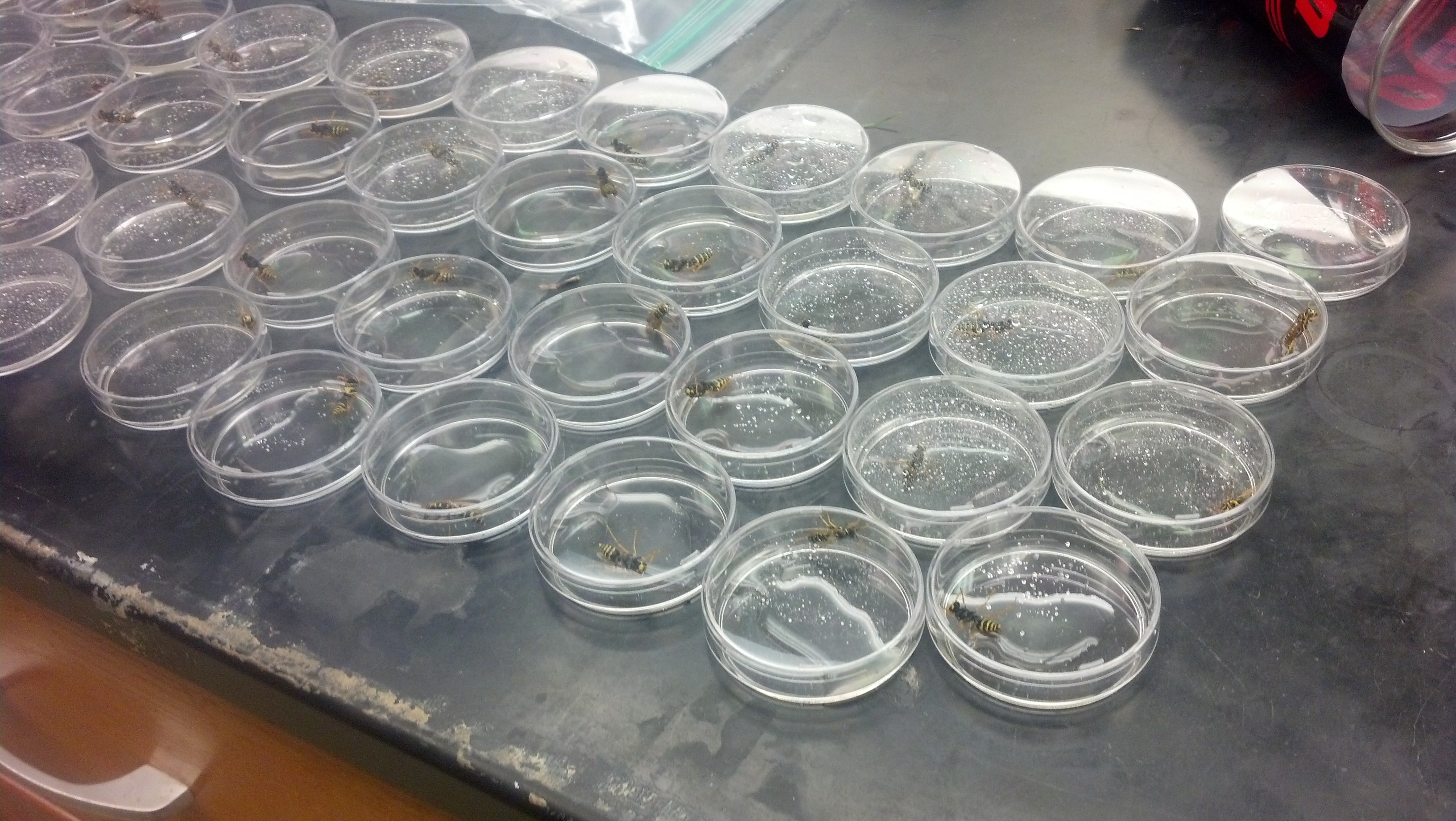


**Figure S1**. Inoculation of *P. dominulus* with the sour rot community *A. aceti* and *Sc. pombi*.


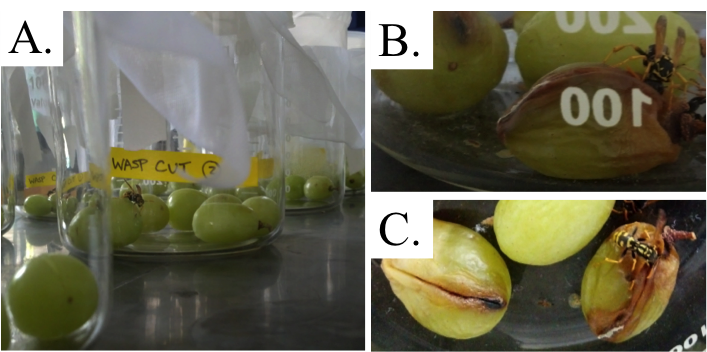


**Figure S2.** Sour rot inoculated *P. dominulus* foraging on grape berries in the rot dispersal assay. (A.) *P. dominulus* feeding on grape berriess on day one of the assay. (B.) and (C.) *P. dominulus* feeding on grape berries on day 13 of the assay. Note that the head of the wasp is fully within the injured flesh of the berry when foraging.


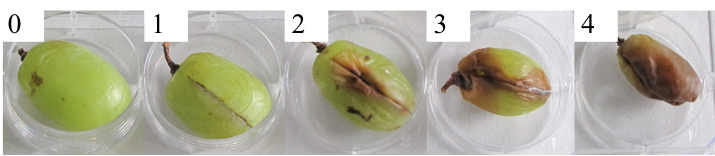


**Figure S3**. Qualitative guide used to assess overall grape berry disease score (0-4).


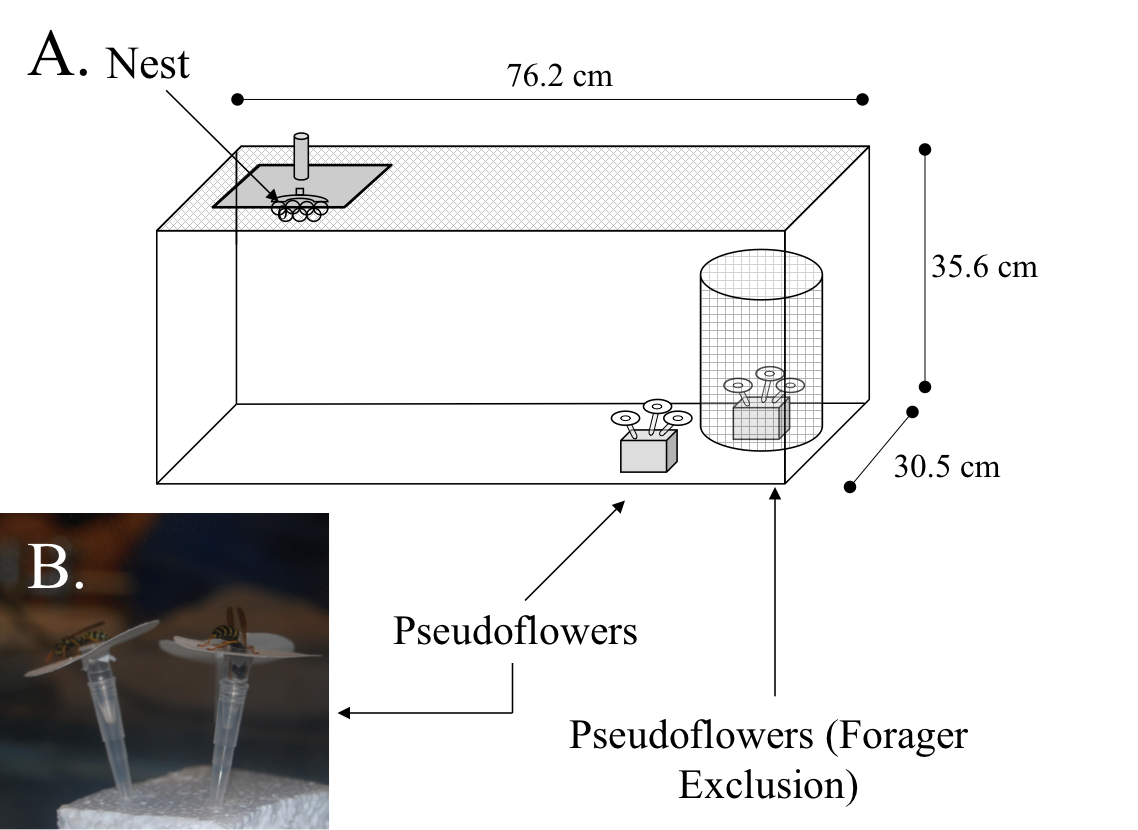


**Figure S4.** (A.) Schematic of sterile foraging enclosure to assess microbial dispersal (B.) *P. dominulus* foraging on the pseudoflowers.

**Detailed methods for dispersal assay:**

The glass enclosures measured 76.2 cm x 35.6 cm x 30.5 cm and were topped with screen netting. The enclosures were cleaned with a 20% sodium hypochlorite solution prior to testing to reduce the amount of transient microbes. One nest was added to one side of each enclosure (with nest sizes > five adults). On the other side of the enclosure, two clusters of ‘pseudo-flowers’ were placed to induce foraging. Pseudoflowers were constructed from Whatman filter paper 40 (GE Healthcare Life Sciences, Piscataway, NJ) cut in disks with a diameter of 3.8 cm to approximate the size of environmentally relevant flowers visited by these wasps (Lauri et al. 1996). In the center of the disk, a standard 200 μl pipet tip (Fisher Scientific) held the uncapped tube of a 200 μl MicroAmp® microcentrifuge tube (Applied Biosystems). The pseudo-flowers were sterilized via autoclaving and secured in polystyrene foam mounts in the enclosures in groups of three flowers. To each flower, 200 μl of a sterile, 20% sucrose solution was added to provide bait for foragers. One group of the flowers was covered with a netting to prevent forager visits and serve as a negative control. Paper wasps were maintained in this foraging enclosure for 24 h and watched to assure at least one wasp had foraged on at least one pseudoflower. Any nests where foraging was not observed after 24 h were not included in the analysis. After the 24 h, all pseudoflowers were aseptically removed and the filter paper pseudopetals were removed from the pseudoflowers. The pseudopetals were imprinted face-down on PDA. After 72 h of growth in the dark at approximately 22°C, the number of macroscopic colonies deposited per square centimeter of pseudo-petal area was recorded. The assay was replicated 11 times using 11 different nests. We statistically analyzed whether pseudo-flowers in the presence of wasps had more microbes than forager-excluded pseudo-flowers using paired t-tests in R (R Core Team 2012). The procedure was validated by replicating the assay without foraging wasps to confirm that the forager exclusion screen did not affect the number of airborne spores randomly deposited on the pseudo-flowers (n = 10).


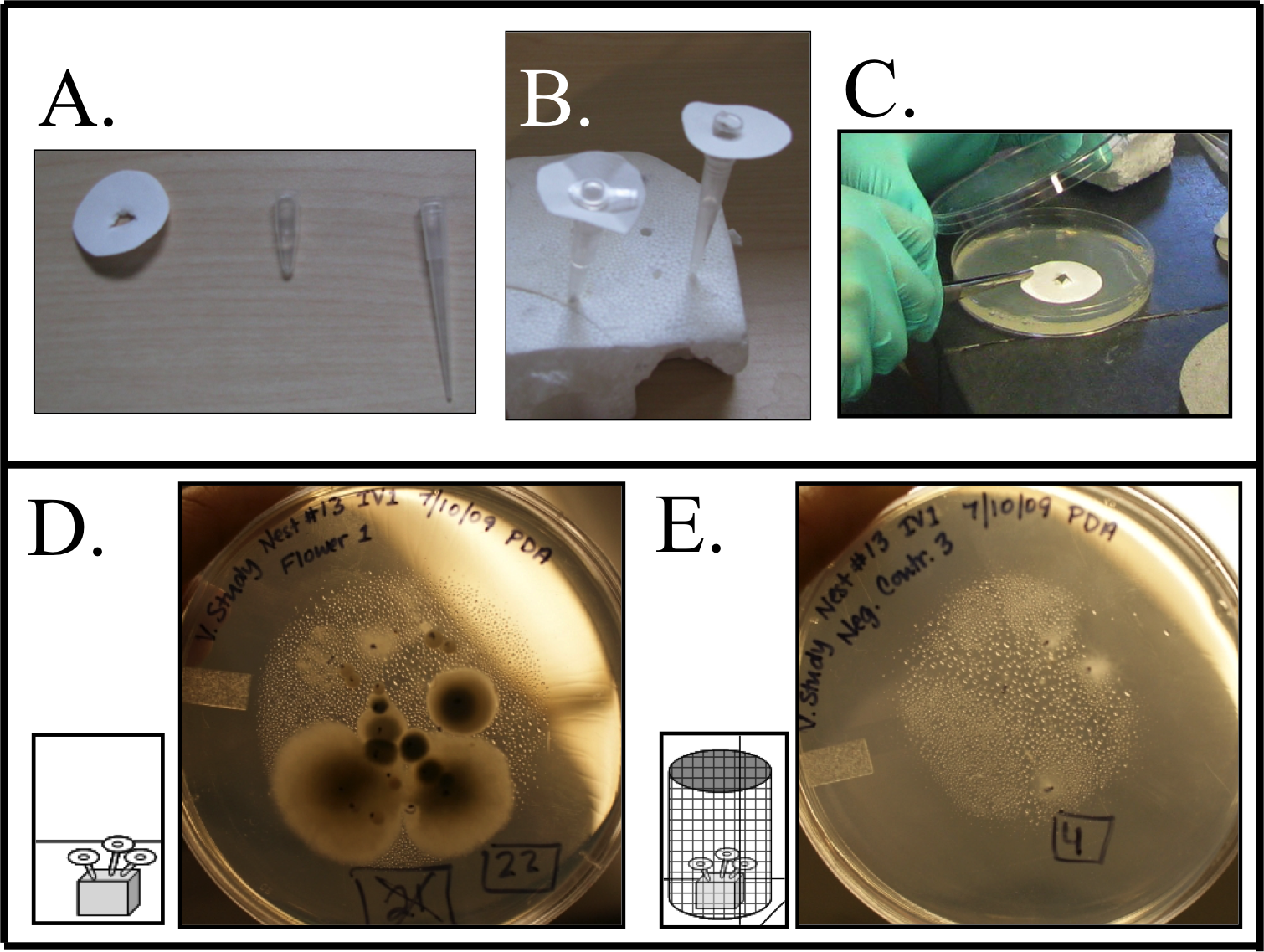


**Figure S5.** Dispersal assay methodology and representative dispersed microbial growth: (A.) Pseudoflower constituents. (B.) Pseudoflowers following sterilization. (C.) Pseudoflowers imprinted on agar nutrient medium following wasp foraging. (D.) Representative plate of a pseudoflower visited by *P. dominulus*. (E.) Representative plate of a pseudoflower with wasps excluded.


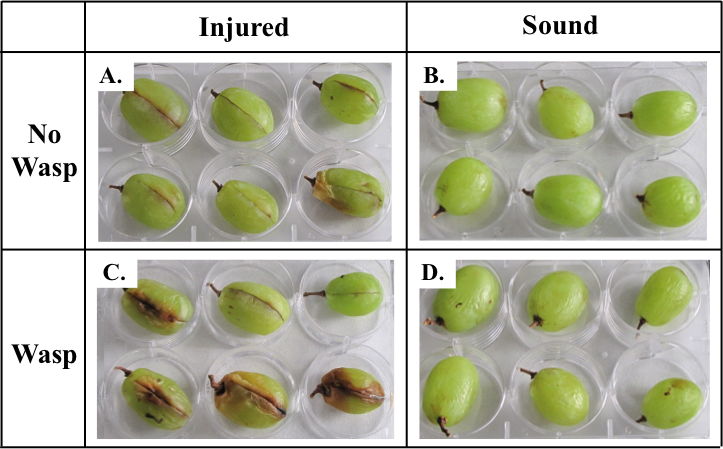


**Figure S6** Representative trials of grape berries maintained in the presence or absence of sour rot inoculated *P. dominulus* for 13 days. (A.) Injured grapes no wasps, (B.) Uninjured grapes no wasps, (C.) Injured grapes with wasps, (D.) Uninjured (Sound) grapes with wasps.


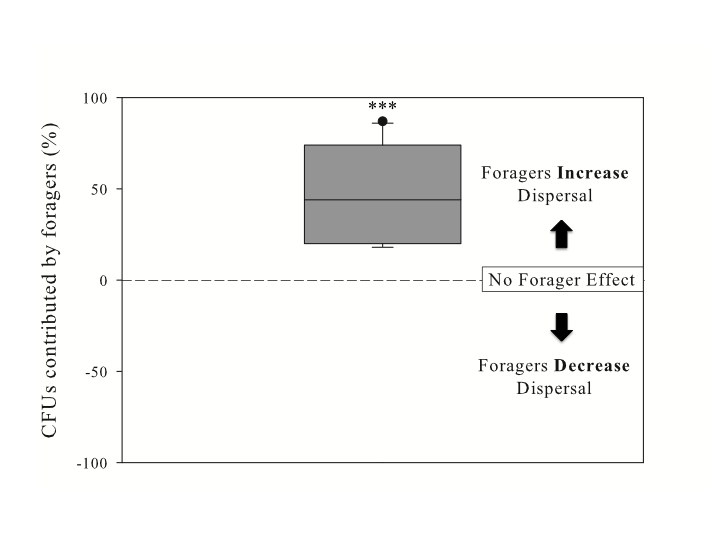


**Figure S7.** Percent increase in dispersed viable microbes (CFUs) on pseudoflowers visited by foraging wasps relative to forager-excluded pseudoflowers. Data are normalized to paired treatment colony forming units dispersed in the absence of foraging wasps (paired t-test, *t* = 7.12, df = 10, p < 0.0001).

**Supplemental Video**

**Video_Madden.mov available at FigShare:**

[**https://dx.doi.org/10.6084/m9.figshare.4551145.v1**](https://dx.doi.org/10.6084/m9.figshare.4551145.v1)

**Video S1.** Supplemental video of uninoculated wasps foraging on grape berries.

**Supplemental Table**

| **Table S1. Identification of fungal strains isolated from grape berries with black mold based on ITS1-5.8S-ITS2 rDNA sequencing.** | | | |
| --- | --- | --- | --- |
| Treatment | Isolate (GenBank ID) | Putative Identification | Match to GenBank record |
| Injured Control | C6F (KF737865) | *Aspergillus niger* | 99% to KJ365326.1 |
| Injured with Wasps | WC3E (KF737864) | *Aspergillus niger* | 99% to KJ365326.1 |
| Injured with Wasps | WC5B (KF737863) | *Aspergillus niger* | 99% to KJ365326.1 |
| Injured with Wasps | WC5F (KF737862) | *Aspergillus niger* | 99% to KJ365326.1 |
|  |  |  |  |
